# Takayasu’s Arteritis: A Special Case Report and Review of the Literature

**DOI:** 10.3390/medicina60030456

**Published:** 2024-03-09

**Authors:** Paloma Moisii, Irina Jari, Alexandru Gratian Naum, Doina Butcovan, Grigore Tinica

**Affiliations:** 1 1st Medical Department, “Gr. T. Popa” University of Medicine and Pharmacy, 16 Universitatii Street, 700115 Iasi, Romania; 2 “Promedicanon” Cardiology Office, 15 Prisacii Valley, Valea Lupului, 707410 Iasi, Romania; 3 2nd Surgical Department, “Gr. T. Popa” University of Medicine and Pharmacy, 16 Universitatii Street, 700115 Iasi, Romania; irinajari25@gmail.com; 4 “Sf. Spiridon” Emergency Hospital, Radiology and Medical Imaging Clinique, 1st Independentei Avenue, 700111 Iasi, Romania; 5 2nd Morphofunctional Sciences Department, Biophysics and Medical Physics, “Gr. T. Popa” University of Medicine and Pharmacy, 16 Universitatii Street, 700115 Iasi, Romania; alexandru.naum@gmail.com; 6 “Neolife” Medical Center, 52 Carol I Avenue, 700503 Iasi, Romania; 7 1st Morpho-Functional Sciences Department, “Gr. T. Popa” University of Medicine and Pharmacy, 16 Universitatii Street, 700115 Iasi, Romania; butcovan@yahoo.com; 8 Pathology Department, “Prof. Dr. George Georgescu” Institute of Cardiovascular Diseases, 50 Carol I Avenue, 700503 Iasi, Romania; 9 1st Surgery Department—Cardiac Surgery, “Gr. T. Popa” University of Medicine and Pharmacy, 16 Universitatii Street, 700115 Iasi, Romania; grigore.tinica@umfiasi.ro; 10 Cardiac Surgery Department, “Prof. Dr. George Georgescu” Institute of Cardiovascular Diseases, 50 Carol I Avenue, 700503 Iasi, Romania

**Keywords:** Takayasu’s arteritis, angiography, clinical criteria, morphopathology

## Abstract

*Background*: Takayasu’s arteritis is a rare type of vasculitis with severe complications like stroke, ischemic heart disease, pulmonary hypertension, secondary hypertension, and aneurysms. Diagnosis is achieved using clinical and angiographic criteria. Treatment is medical and surgical, but unfortunately, the outcome is limited. *Case presentation*: A 34-year-old Caucasian woman had an ischemic stroke (2009). She was diagnosed with Takayasu’s arteritis and received treatment with methotrexate, prednisolone, and antiplatelet agents, with a mild improvement in clinical state. After 6 years (2015), she experienced an ascending aorta aneurysm, pulmonary hypertension, and mild aortic regurgitation. Surgical treatment solved both the ascending aorta aneurysm and left carotid artery stenosis (ultrasound in 2009 and computed tomography angiogram in 2014). Morphopathology revealed a typical case of Takayasu’s arteritis. Tumor necrosis factor inhibitors (TNF inhibitors) were prescribed with methotrexate. At 48 years old (2023), she developed coronary heart disease (angina, electrocardiogram); echocardiography revealed severe pulmonary hypertension, and angiography revealed normal coronary arteries, abdominal aorta pseudoaneurysm, and arterial–venous fistula originating in the right coronary artery with drainage in the medium pulmonary artery. The patient refused surgical/interventional treatment. She again received TNF inhibitors, methotrexate, antiplatelet agents, and statins. *Conclusions*: This case report presented a severe form of Takayasu’s arteritis. Our patient had multiple arterial complications, as previously mentioned. She received immunosuppressive treatment, medication targeted to coronary heart disease, and surgical therapy.

## 1. Introduction

Takayasu’s arteritis (TA) is a specific vasculitis that is diagnosed and treated by teams. These teams comprise a rheumatologist, an imaging specialist, a cardiologist, an interventional cardiologist, a cardiac surgeon, and a pathologist. The disease affects large arteries via granulomatous inflammation. Stenosis, aneurysmal dilation, and occlusion are observed in advanced stages [1]. Stenosis and occlusion are more common in Europe, the United States, and Japan; aneurysms are more prevalent in India, Thailand, Mexico, and Africa [2]. Medical treatment is addressed to most patients, and selected cases can benefit from surgical interventions. Death can occur due to cerebral thrombosis and hemorrhage, myocardial infarction, aneurysm rupture, or renal or heart failure. Takayasu patients with active disease and surgery requirement usually evolve to arterial inflammation at another location [3]. The first case was presented by Mikito Takayasu in 1908 at the Annual Meeting of the Japan Ophthalmology Society. He noticed arteriovenous anastomosis around the papilla of a 21-year-old woman [4]. Takayasu did not diagnose any other medical issues in his patient, but his name is still applied to this disease. After Dr. Takayasu, ophthalmologists also noticed radial artery involvement, and the disease was considered a vasculitis. The correlation between retinal artery involvement in TA and large artery damage are still useful in clinical practice, as they are noninvasive. Poignet et al. assessed microaneurysms in the central retinal artery via Doppler ultrasound imaging, and computed tomography angiography evaluated supraaortic stenosis. The authors discovered a significant correlation between the number of retinal artery microaneurysms and large artery stenosis in TA. Furthermore, the number of retinal artery microaneurysms can be considered a prognostic factor in TA [5]. The American College of Rheumatology (ACR) established six diagnosis criteria for TA in 1990, including onset before 40 years old; superior limb claudication; diminished pulsation in the brachial artery; at least 10 mmHg difference in systolic blood pressure between the right and left arms; bruit in abdominal aorta/subclavian arteries; and the narrowing or occlusion of the aorta or main branches upon angiography. At least three of these six criteria constituted a definite diagnosis of this disease [6]. In 2022, the ACR updated these criteria as follows in Table 1.

Each pathological finding receives a score from 1 to 3. A total score ≥ 5 constitutes a definite diagnosis of this disease [7]. Nowadays, clinicians can use vascular imaging. The first was classical angiography was an invasive technique [8]. Computed tomography angiography (CTA) and magnetic resonance arteriography (MRA) are noninvasive imaging techniques. The first choice is MRA, and CTA and ultrasound are alternatives [9]. Fluorodeoxyglucose positron emission tomography (FDG-PET) measures vascular inflammation. FDG-PET calculates the integrated disease activity index [10]. Morphopathology plays a major role in a better understanding of disease pathogenesis. Fragments of the affected arteries can reveal the involvement of all layers, including adventitia inflammation, the destruction of media elastic tissue, and neovascularization of the intima and media [11]. Macrophages participate beyond all stages of arterial inflammation, as well as in arterial remodeling [12]. Triggers for this autoimmune disorder can be tuberculosis in different locations [13]. Differential diagnosis is conducted with arteritis from syphilis, systemic erythematous lupus, rheumatoid arthritis, sarcoidosis, and Marfan syndrome [14]. Treatment is medical and/or interventional or surgical. Steroids and/or methotrexate are first-line medications [15]. Tumor necrosis factor (TNF) inhibitors are prescribed to refractory/severe TA patients. TNF inhibitors interact with interleukin 6 (IL 6) [16]. Janus kinase inhibitors (JAKinibs) modulate signaling for multiple interleukins, including 2, 6, 12, and 23 (not only IL 6 like TNF inhibitors) [17]. Disease activity can be assessed using clinical indices and biomarkers [18]. Interventional methods can usually solve arterial stenosis and arterial dilation. Surgical therapy is applied for aneurysms, aortic regurgitation, and arterial stenosis or dilation when interventional treatment is not appropriate [19]. The prognosis is uncertain, with relapses and possible complications. Stroke, aortic dissection/rupture, myocardial infarction, severe pulmonary hypertension, the side effects of immunosuppressive therapy, cancers, and infections are common complications [20,21]. Biologic therapy and real improvement in long-term outcomes of TA represent a gap in the research. The aim of this case report is to present special manifestations in this vasculitis, such as an interesting mixture of stable and active disease, and severe complications following disease onset. 

## 2. Case Presentation

A 48-year-old Caucasian woman was referred to the cardiologist because of increasing prolonged crises of the angina pectoris during the previous 2 months. Her medical history began in 2008, when she was 33 years old; she was diagnosed and treated for pulmonary tuberculosis. The next year, she was diagnosed with right hemiparesis associated with fever, weight loss of 5 kg in 1 month, malaise, and increased acute phase reactants levels. The erythrocyte sedimentation rate level was 55 mm/h, and the C reactive protein level was 6 mg/L. She was hospitalized at the neurology department, and a clinical examination revealed a total score of six, according to the ACR 2022 criteria for TA [7] (see Table 2).

**Figure 1:**
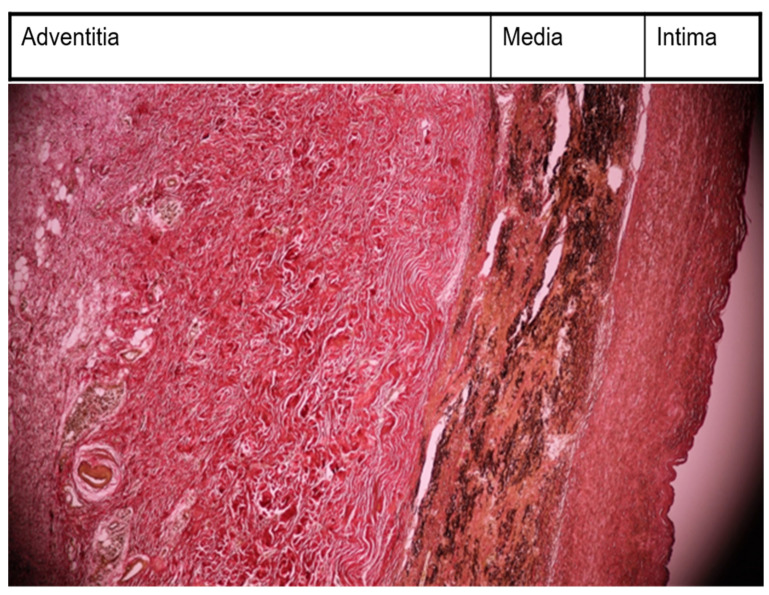
Morphopathological examination—thickened intima, with fibrosis; alteration of elastic structure of media; very thickened adventitia collagenized (EVG, ×10).

**Figure 2:**
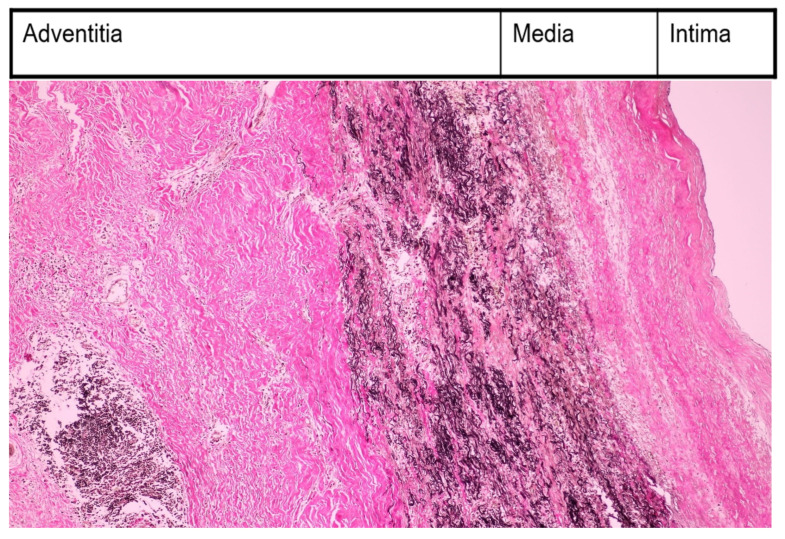
Morphopatological examination—inflammatory infiltration with monocytes in media and adventitia (EVG, ×200).

The suspicion of Takayasu arteritis (an ACR score ≥ 5) was confirmed by the rheumatologist. A Doppler ultrasound of the cervical arteries revealed a critical stenosis (75%) in the left carotid artery, but the patient refused interventional/surgical therapy. She received prednisolone (PDN) at 50 mg/day (1 mg/kg) by mouth, methotrexate (MTX) at 20 mg weekly, intravenously, and aspirin at 100 mg/day by mouth. Immunosuppressive therapy doses were established by the rheumatologist. PDN at 50 mg was maintained for 2 weeks; beginning with the 3rd week, the dose of PDN was slowly decreased by 5 mg every week. After 8 weeks, the maintenance dose of PDN at 20 mg daily was achieved. This dose was maintained for the following 2 years. Intravenously, MTX was administered for 2 weeks at 20 mg weekly. Beginning with the third week, we reduced the MTX dose by 5 mg every week. After 5 weeks, the maintenance dose of 5 mg of MTX weekly was reached, and the route of administration was by mouth. MTX was administered for the following 2 years, together with PDN. Long-term treatment with the antiplatelet drug, aspirin, at 100 mg/day was recommended. Regular monthly checkups were necessary. These checkups included a clinical examination that focused on glycemia, complete blood count, and renal and liver function. The only side effect was nausea. The general symptoms vanished after 1 month. Neurological recovery was almost complete after 3 months, and acute-phase reactant levels normalized after 1 month. After 6 years of disease remission, in 2015, when she was 40 years old, the patient was hospitalized at the cardiology department with malaise, sweating, dyspnea after minor efforts, and palpitations. Echocardiography revealed moderate pulmonary hypertension. Computed tomography angiography confirmed the critical stenosis of the left carotid artery at 85% (already discovered 6 years ago via Doppler ultrasound) and revealed an ascending aorta aneurysm. The cardiac surgeon removed the ascending aorta aneurysm via open surgical repair and inserted a graft in its place. He also performed a left carotid endarterectomy, removed the stenotic area, and inserted a graft in its place. The ascending aorta aneurysm removed by the cardiac surgeon was cut into fragments in the histology laboratory. These fragments were placed in 10% formalin solution, embedded, and cut with a microtome. Anatomical fragments from the aorta had modifications typical of Takayasu’s arteritis (see Figure 1 (EVG = elastic Van Gieson discoloration) and Figure 2).

In our case, the morphopathologist’s verdict was that the patient was in the chronic phase with acute relapses, so we decided that immunosuppressive treatment was still necessary.

We gave her Infliximab, a tumor necrosis factor inhibitor (TNF inhibitor) via 90 min intravenous perfusion (3 mg/kg = 150 mg), according to the rheumatologist’s indications. The second dose of Infliximab was given 2 weeks after the first dose, and the third dose of TNF inhibitor was provided to the patient 6 weeks after the first dose. In the following 2 years, she received 150 mg of Infliximab every 6 weeks. MTX at 5 mg/day was administered with the Infliximab during the following 2 years, as advised by the rheumatologist. Infliximab is a monoclonal antibody that neutralizes tumor necrosis factor-alpha and diminishes inflammation [22]. The adverse effects were minor and included nausea and muscle stiffness. Regular monthly checkups for a clinical examination, renal and hepatic function, and complete blood count was recommended.

**Figure 3:**
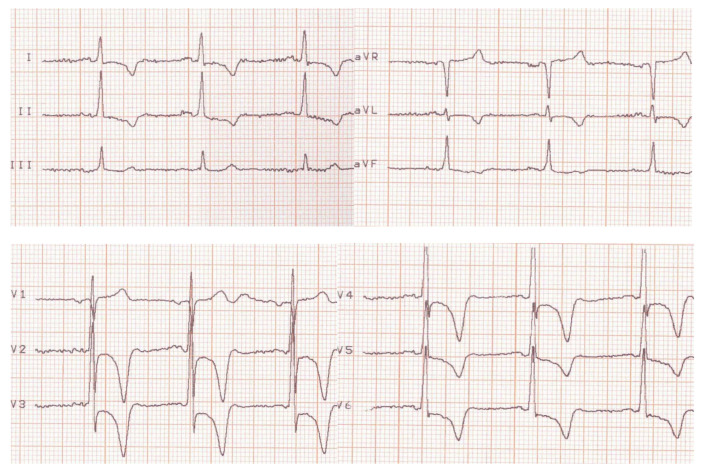
Current electrocardiogram (before treatment).

Her condition improved until 2023, when she experienced a severe and prolonged angina pectoris crisis. A current electrocardiogram revealed ST segment depression in the DI, avL, and V2–V6, with giant negative T waves. These electrocardiogram findings were novel (see Figure 3).

Emergency blood tests were recommended because of the clinical description of severe angina pectoris and profound alterations seen in the electrocardiogram, which suggested a possible non-STEMI. Table 3 reflects normal values for biomarkers (ASAT and ALAT < 40 IU/L; CPK = 30–135 IU/L in females; troponin T and I < 14 ng/L); therefore, an acute myocardial infarction was excluded.

**Figure 4:**
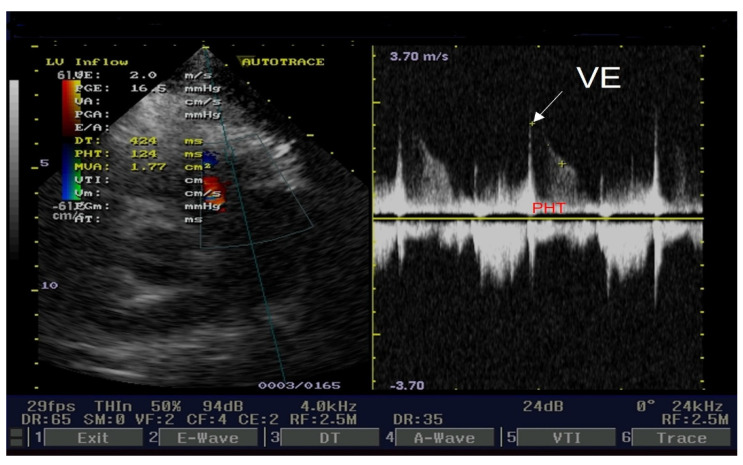
Echocardiography short axis view: PHT for pulmonary regurgitation.

The cardiologist prescribed beta-blockers (Nebivolol, 5 mg/day), antiplatelet agents (aspirin, 100 mg/day), statins (Atorvastatin, 20 mg/day), and angiotensin-converting enzyme inhibitors (Perindopril, 5 mg/day). The patient’s angina and electrocardiographic alterations diminished. Transthoracic echocardiography revealed signs of severe pulmonary hypertension. PHT for pulmonary regurgitation is an important parameter for a correct approach to pulmonary hypertension severity. Our cardiology ultrasound machine was a Fukuda Denshi 850-XTD. Figure 4 illustrates PHT determination. The left side of the figure presents three physics parameters. The first one is the end-diastole velocity (VE = 2 m/s). The second one is the pressure gradient between the right ventricle and the pulmonary artery trunk end-diastole (PGE = 16.5 mmHg). The third is pressure half time, which is the time necessary for PGE to halve itself (PHT = 124 ms). PHT ranges ≤ 150 ms represent severe pulmonary hypertension. Also, the left side of this figure presents color flow for pulmonary regurgitation PR and Doppler sample volume. The right side of the figure presents the PR envelope with end-diastole velocity (white arrow) and PHT (red capital letters). 

**Figure 5:**
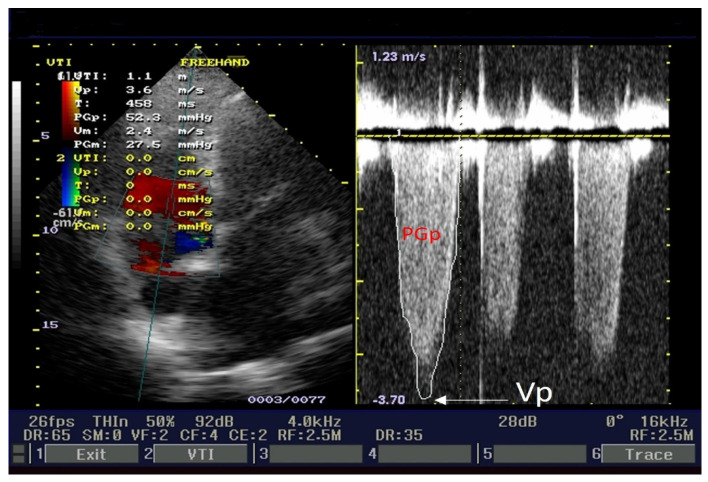
Echocardiography apical 4-chamber view: pulmonary artery systolic pressure determination.

Our patient revealed severe tricuspid regurgitation on a color Doppler. The left side of Figure 5 illustrates an apical four-chamber view, with tricuspid regurgitation and pulsed Doppler sample volume. Peak velocity (Vp = 3.6 m/s), peak pressure gradient (PGp = 52.3 mmHg), median velocity (Vm = 2.4 m/s), and median pressure gradient (PGm = 27.5 mmHg) are displayed on the left side of Figure 5. These parameters are velocities and gradients created by tricuspid regurgitation. The right side of Figure 5 presents the tricuspid regurgitation envelope (red capital letters label PGp). We estimated right atrial pressure at 10 mmHg because the inferior cava vein (ICV) diameter = 21 mm, and its collapsibility index was ≥50%. Pulmonary artery systolic pressure was 52 mmHg + 10 mmHg = 62 mmHg; ranges ≥ 55 mmHg represent severe pulmonary hypertension.

**Figure 6:**
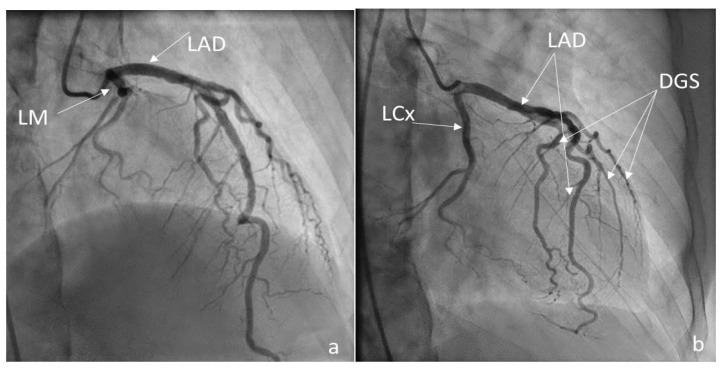
(**a**,**b**) Coronarography RAO views LM = left main trunk, LAD = left anterior descending, LCx = circumflex, Dgs = diagonals.

**Figure 7:**
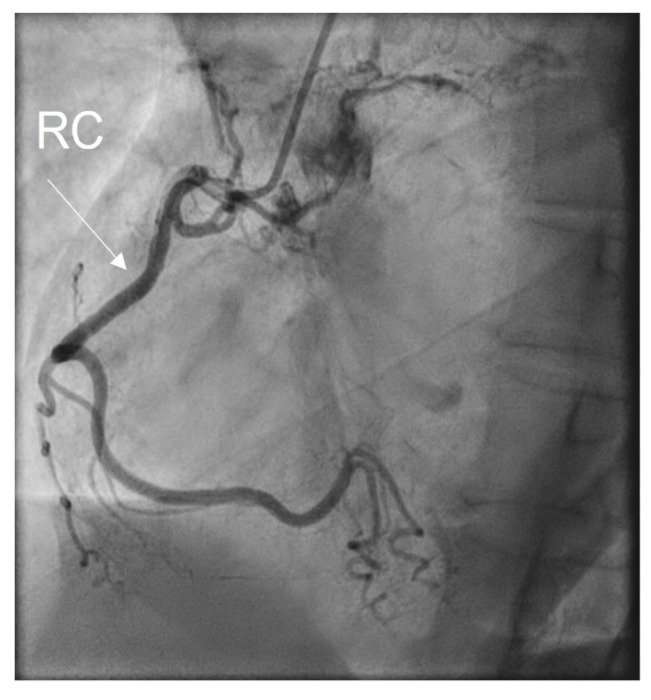
Coronarography LAO view: RC = right coronary artery.

A coronarography was recommended, and the large coronary arteries were found to be permeable, without significant lesions: see Figure 6a,b and Figure 7. Angina and electrocardiographic alterations were explained by microvascular lesions (female, hypercholesterolemia) and a coronary steal mechanism, as described in Takayasu’s arteritis. Unfortunately, we did not have the capability of obtaining special imaging in microvascular coronary disease. This can be assessed using coronary flow reserve (CFR) measurement via noninvasive or invasive techniques. Noninvasive methods are PET, as the gold standard, MRI, and transthoracic echocardiography (TTE) for LAD. The invasive method for CFR measurement is the assessment of an intracoronary Doppler-pressure wire [23,24]. Figure 6a,b are RAO views of the permeable left coronary tree, including the left main trunk, the left anterior descending artery (LAD), the left circumflex artery (LCx), and diagonals. Figure 7 is a LAO view of the permeable right coronary tree.

**Figure 8:**
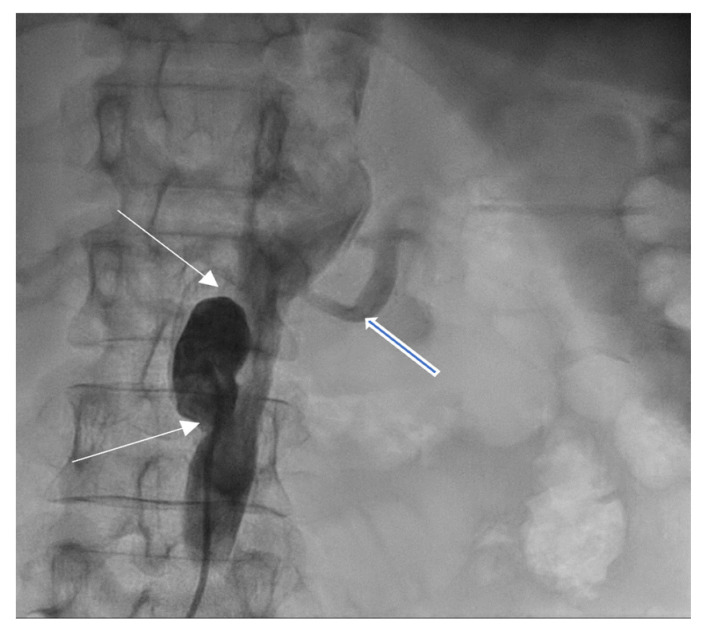
Abdominal aortography, antero-posterior view—abdominal aorta pseudoaneurysm (white arrows) below left renal artery (open blue arrow).

**Figure 9:**
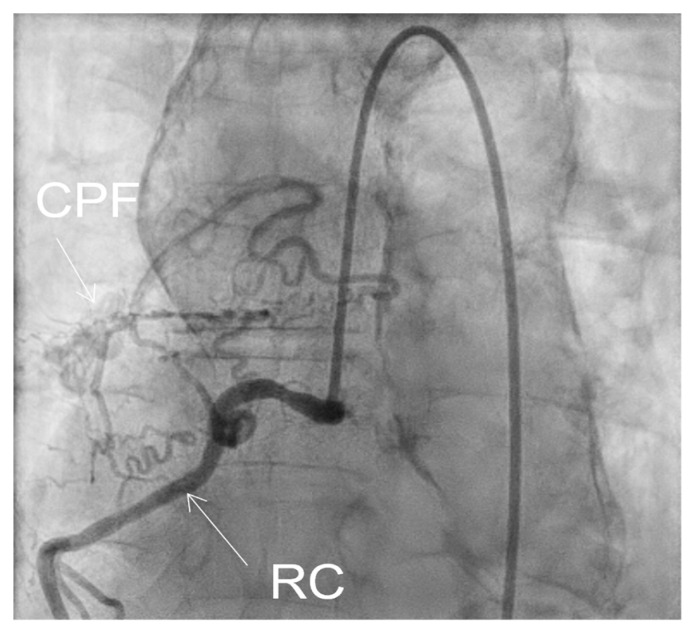
Fistula CPF = coronary–pulmonary fistula, originating in right coronary RC.

Coronarography was followed by conventional aortography of an abdominal aorta pseudoaneurysm (Figure 8) and an arterial–venous fistula originating in the right coronary artery with drainage in the medium pulmonary artery (Figure 9).

Table 4 illustrates the current diagnostic criteria, according to ACR EULAR 2022 [7]. Our patient registered a total score of eight points. The affected arterial territories included the superior limbs and the abdominal aorta.

**Figure 10:**
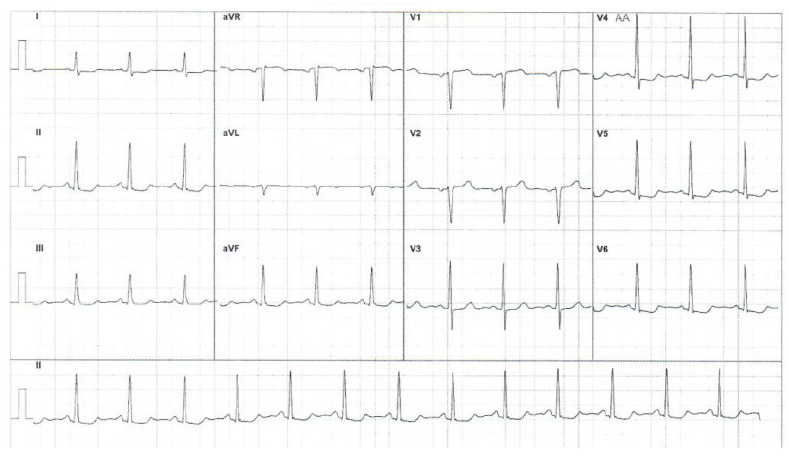
Electrocardiogram after specific treatment.

The patient refused a new operation for the abdominal aorta pseudoaneurysm and fistula originating in the right coronary artery with drainage in the medium pulmonary artery. The rheumatologist recommended immunosuppressive treatment with TNF inhibitors (Infliximab) at 150 mg every 8 weeks (intravenous perfusion, 90 min) and MTX at 20 mg weekly and intravenously for the following 2 years. Regular checkups, including renal and hepatic function and complete blood count, every 2 months was recommended. The cardiologist recommended long-term administration of beta-blockers (Nebivolol at 5 mg/day), antiplatelet agents (aspirin at 100 mg/day), statins (Atorvastatin at 20 mg/day), and angiotensin-converting enzyme inhibitors (Perindopril at 5 mg/day). Regular checkups, including electrocardiograms and echocardiograms, every 6 months was recommended. A moderate improvement was recorded after these medications, including diminishing frequency and severity of the angina crisis. Electrocardiogram pathological findings also diminished, and only minor ST depression was noticed in DII, DIII, avF, and V4–V6 (see Figure 10). 

## 3. Discussion

The case had the following pathological findings: stenosis on a major branch of the aorta, the left carotid artery; general symptoms (fever, malaise, weight loss); an ascending aorta aneurysm; pulmonary hypertension; angina pectoris; an abdominal aorta pseudoaneurysm; and a fistula originating in the RCA with drainage in medium pulmonary artery. The stenosis, aneurysm, and pseudoaneurysm we observed were typical for TA and are a consequence of large artery damage. Pulmonary artery involvement in TA is often associated with coronary heart disease, as Mykoyama et al. observed [25]. Our patient presented with angina pectoris (symptoms, electrocardiogram alterations) and pulmonary hypertension. We considered that severe pulmonary hypertension was explained by the following two mechanisms for our patient: direct involvement of pulmonary arteries and a coronary–pulmonary fistula. This fistula provided a left-to-right shunt from the right coronary artery to the medium pulmonary artery. Pathological findings are important, but disease activity leads the therapy in TA. Kermani et al. presented, at an ACR convergence in 2023, two interesting indices for disease activity in TA, which included the vasculitis damage index and the large vessel vasculitis index of damage [26]. The authors posited that these indices would be useful in clinical trials involving TA. Their study included 350 patients with TA from North America and Turkey. They noticed arterial damage in 80% of patients from the first medical examination (our case history began with left carotid artery stenosis and its complication from the first medical visit). During follow-up, 40% of Kermani et al.’s patients experienced the addition of cardiovascular involvement (during 14 years of follow-up, our patient experienced the addition of the involvement of the ascending aorta, abdominal aorta, and pulmonary arteries). There are several markers like autoantibodies, adipokines, cytokines with great importance in diagnosis, disease activity, and therapy [27]. Their presence and ranges could be useful for an earlier diagnosis, appropriate follow-up, and outcome evaluation. We used morhopathological findings for establishing disease activity. Pathological findings based on histopathology in our case report included the following: thickened intima with fibrosis; alteration of elastic structure of media; thickened adventitia (Figure 1); and inflammatory infiltration with monocytes in media and adventitia (Figure 2). This is a peculiar aspect of our case, which had an interesting mixture of acute (inflammatory infiltration) and chronic (fibrosis) patterns, despite specific treatments. The implication of monocytes and cytokines related to the cells was detailed by de Aguier et al. [28]. The authors measured monocyte-related chemokine levels. These levels were higher in active TA than in stable disease. Active disease was associated with monocytosis. In our case report, we decided that monocyte infiltration in media and adventitia (fragments obtained from ascending aorta aneurysm) suggested an active disease. Differential diagnosis for our case is illustrated in Table 5. 

Parakh and Yadev described three phases in TA, which include systemic features, arterial inflammation, and stenosis [29]. The simultaneous presence of two stages is another peculiar aspect of our patient. The first phase featured general symptoms, and the third phase featured left carotid artery stenosis. A triggering factor for TA is tuberculosis; many patients diagnosed with this vasculitis have also had tuberculosis in their medical history [30], and this was the situation in our case, too. Li et al. reviewed 30 studies with 5548 patients. The authors noticed a 31% prevalence of tuberculosis infection among TA patients. These patients had tuberculosis in their past medical history, or an active/latent tuberculosis infection was discovered concomitantly with vasculitis. The gold-standard imaging techniques for this disease are MRA and CTA [31]. The clinicians will select the imaging according to radiologist expertise and local possibilities; CTA is less expensive and usually provides better resolution than MRA. We utilized CTA for the diagnosis of ascending aorta aneurysm. Arita et al. outlined current immunosuppressive treatments for TA, which include modern DMARDs and JAKinibs [32]. Joseph et al. published updated protocols for TA treatment [33]. Glucocorticoids are still first-line medications, as the remission rate is around 60%, but side effects are frequent. Disease-modifying antirheumatic drugs (DMARDs) are divided into two categories, including synthetic and biologic. DMARDs can be administered with glucocorticoid, or two DMARDs from different categories can be administered together. Synthetic DMARDs are conventional, like MTX and targeted, named JAKinibs, like tofacinib. Biologic DMARDs target interleukin-6 (IL-6), like tocilizumab or are TNF- inhibitors, like Infliximab. Our patient received PDN at 50 mg/day by mouth, MTX at 20 mg/week intravenously, and aspirin at 100 mg/day when she was first diagnosed (2009). The decision-making process was according to international guidelines for first-line TA medication [34]. In the case presentation section, we detailed the decreasing doses for PDN and MTX, as well as the maintenance doses. This treatment was administered for 2 years, and disease remission was recorded for the following 6 years. Misra et al. posited the following: biologic therapy is recommended in refractory TA; biologic DMARDs are more effective than synthetic DMARDs; and angiographic stabilization of arterial involvement is more significant after biologic DMARDs. The authors observed that relapses and side effects are more common after the administration of biologic DMARDs, even if positive effects are superior in biologic DMARDs compared with synthetic DMARDs [35]. Intravenously administered Infliximab at 150 mg replaced PDN in 2015 for our patient. MTX was administered at 5 mg/day by mouth, in addition to Infliximab. We gave her these medications for the following 2 years, and remission lasted for 8 years. This decision was made because relapse and severe complications occurred, including ascending aorta aneurysm and pulmonary hypertension [19]. The side effects of the drugs were minor in our case and included muscle stiffness and nausea. The cardiac surgeon placed grafts on the patient’s left carotid artery and aorta. The second relapse occurred in 2023 and involved severe angina pectoris, an abdominal aorta pseudoaneurysm, and a coronary–pulmonary fistula. She received treatment with Infliximab at 150 mg every 8 weeks intravenously and MTX at 20 mg weekly and intravenously during the following 2 years, according to the updated international ACR guidelines [33]. She also received medication for coronary heart disease, as we described in case presentation section. We noticed a mild improvement in the angina pectoris. The presented case depicts a patient with life-threatening complications caused by TA. A multidisciplinary approach is always necessary in TA diagnosis, follow-up, and treatment. Rheumatologists, cardiologists, radiologists, cardiac surgeons, and morphopathologists collaborate to improve the quality of life and outcome for TA patients.

## 4. Conclusions

TA is large vessel vasculitis that can be life-threatening. This case describes a severe type of TA that included stroke, an ascending aorta aneurysm, pulmonary hypertension, an abdominal aorta pseudoaneurysm, angina pectoris, and a coronary–pulmonary fistula. Imaging techniques included Doppler ultrasound, conventional angiography, and computed tomography angiography. The morphopathologist informed us that TA is active. An immunosuppressive treatment was prescribed, and the initial glucocorticoids were replaced by biologic therapy with Infliximab. MTX was administered with each previously mentioned medication. The cardiac surgeon utilized aorta and carotid artery grafts. Our patient had a poor outcome, despite specific TA treatment. Future projects will include the utilization of PET for earlier disease activity identification and prompt intervention.

## Figures and Tables

**Table 1:** Diagnostic criteria according to ACR 2022.

Diagnostic Criteria	Points
The diagnosis of medium-vessel large-vessel arteritis; other vasculitis are excluded; age ≤ 60 years	
Female sex	+1
Limb claudication	+2
Angina pectoris	+2
Arterial bruit	+2
Diminished pulsation of superior limb arteries	+2
Diminished pulsation of carotid artery	+2
Difference ≥ 20 mmHg between right and left arms	+1
Number of arterial territories involved	+1 to +3
Paired artery affected	+1
Abdominal aorta plus renal/mesenteric arteries affected	+3

**Table 2:** Diagnostic criteria (2009).

Diagnostic Criteria	Points	
Female sex	+1	
Left carotid artery bruit	+2	
Diminished pulsation of the superior limb arteries	+2	
Blood pressure difference of 30 mmHg between right and left arms	+1	
	Total	6

**Table 3:** Laboratory findings.

Laboratory Findings	1st Day	2nd Day	3rd Day
AST (IU/L) ^1^	32	27	31
ALT (IU/L) ^2^	25	22	29
CPK (IU/L) ^3^	48	52	58
CPK-MB (IU/L) ^4^	2	1	2
Troponin T (ng/L)	3.1	4.3	1.5
Troponin I (ng/L)	2.6	3.7	1.4

^1^ AST = aspartate aminotransferase; ^2^ ALT = alanine aminotransferase; ^3^ CPK = creatine phosphokinase; ^4^ CPK-MB = creatine phosphokinase myocardial-bound.

**Table 4:** Current diagnostic criteria.

Diagnostic Criteria	Points	
Female sex	+1	
Angina pectoris	+2	
Diminished pulsations of the superior limb arteries	+2	
Blood pressure difference of 30 mmHg between right and left arms	+1	
Number of affected arterial territories	+2	
	Total	8

**Table 5:** Differential diagnosis.

Disease	Pros	Cons
Syphilis	aortitis, ascending aorta aneurysm	negative venereal disease research laboratory (VDRL) and rapid plasma reagin (RPR) tests
Lupus	Aortitis	negative lupus anticoagulant and anticardiolipin antibodies
Rheumatoid arthritis	aortitis	negative rheumatoid factor (RF) and antinuclear antibodies (ANA)
Sarcoidosis	aortic aneurysm	CT scan: no mediastinal and hilar lymphadenopathies
Marfan syndrome	ascending aorta aneurysm	negative Ghent criteria for Marfan syndrome

## Data Availability

Data are available upon request because of privacy restrictions.
